# Event-Driven Immunoprofiling Predicts Return of Disease Activity in Alemtuzumab-Treated Multiple Sclerosis

**DOI:** 10.3389/fimmu.2020.00056

**Published:** 2020-01-31

**Authors:** Katja Akgün, Judith Blankenburg, Michaela Marggraf, Rocco Haase, Tjalf Ziemssen

**Affiliations:** Center of Clinical Neuroscience, University Hospital, Technical University Dresden, Dresden, Germany

**Keywords:** multiple sclerosis, alemtuzumab, treatment response, immunoprofiling, immune cells

## Abstract

**Background:** Alemtuzumab is a highly effective drug for the treatment of multiple sclerosis (MS), characterized by specific patterns of depletion and repopulation. As an induction-like treatment concept, two mandatory infusion courses can inhibit long-term disease activity in the majority of patients, and additional courses can successfully manage subsequent re-emergence of disease activity. Currently, there are no biomarkers to identify patients with re-emergent disease activity requiring retreatment.

**Methods:** In this study, we systematically characterized 16 MS patients commencing alemtuzumab. Clinical parameters, MRI and detailed immunoprofiling were conducted every 3 months for up to 84 months.

**Results:** Alemtuzumab led to significant decrease in clinical disease activity in all evaluated patients. Nine out of 16 patients presented with no evidence of disease activity (NEDA)-3 up to 84 months (“complete-responder”), while 7 patients demonstrated clinical or/and subclinical MRI disease activity and received alemutzumab retreatment (“partial-responder”). In both response categories, all T- and B-cell subsets were markedly depleted after alemtuzumab therapy. In particular, absolute numbers of Th1 and Th17 cells were markedly decreased and remained stable below baseline levels—this effect was particularly pronounced in complete-responders. While mean cell numbers did not differ significantly between groups, analysis of event-driven immunoprofiling demonstrated that absolute numbers of Th1 and Th17 cells showed a reproducible increase starting 6 months before relapse activity. This change appears to predict emergent disease activity when compared with stable disease.

**Conclusion:** Studies with larger patient populations are needed to confirm that frequent immunoprofiling may assist in evaluating clinical decision-making of alemtuzumab retreatment.

## Introduction

The anti-CD52 antibody alemtuzumab (ATZ) exerts its clinical efficacy via a specific pattern of depletion and repopulation of different immune cell subsets in active, relapsing-remitting multiple sclerosis (RRMS) (1–6). Based on expression of CD52 at the cell surface, lymphocyte subtypes are affected differently by the depletion mechanism, which is primarily mediated by antibody and complement-dependent cytotoxicity (2, 7). Previous reports have already demonstrated rapid and dramatic decreases in numbers of lymphocytes and antigen-presenting cells in the hours immediately after ATZ application (8). Specific cellular repopulation patterns appear to be responsible for the long-term efficacy of ATZ that persists years after the last course of therapy (9, 10): T cells remained depleted years after the last ATZ course, but B cells repopulate quickly, even overshooting baseline levels several months after ATZ treatment in the most cases (10–12). These depletion-repopulation kinetics are purportedly responsible for the positive, long-term effects of ATZ on disease activity, and for its established side-effect profile, including secondary autoimmunity (10, 13, 14). Previous evaluations have not been able to make a link between the recovery of selected T and B cells and MS disease activity after ATZ treatment (13, 15).

Two courses of ATZ are used as standard of care resulting in no evidence of disease activity (NEDA)-3 status in the majority of patients (16). But there are some patients that require further courses of ATZ due to emergent disease activity (6). Until now, it has not been possible to identify these patients in advance, and therefore the optimal period of retreatment has been defined. Long-term data are essential to understand and define immunological profiles that can identify re-emergent of clinical or MRI disease activity in order to develop and modify individual retreatment strategies.

Here we present a systematic analysis of clinical and immunological parameters in a cohort of ATZ-treated and re-treated RRMS patients. Our innovative, time-synchronous analysis is based on an individualized, event-driven approach linking onset of clinical disease activity and ATZ retreatment with immunoprofiling. Using this event-driven approach, we aimed to identify disease activity-related immunological patterns in ATZ-treated patients.

## Methods

### Patients and Study Approval

**Figure 1:**
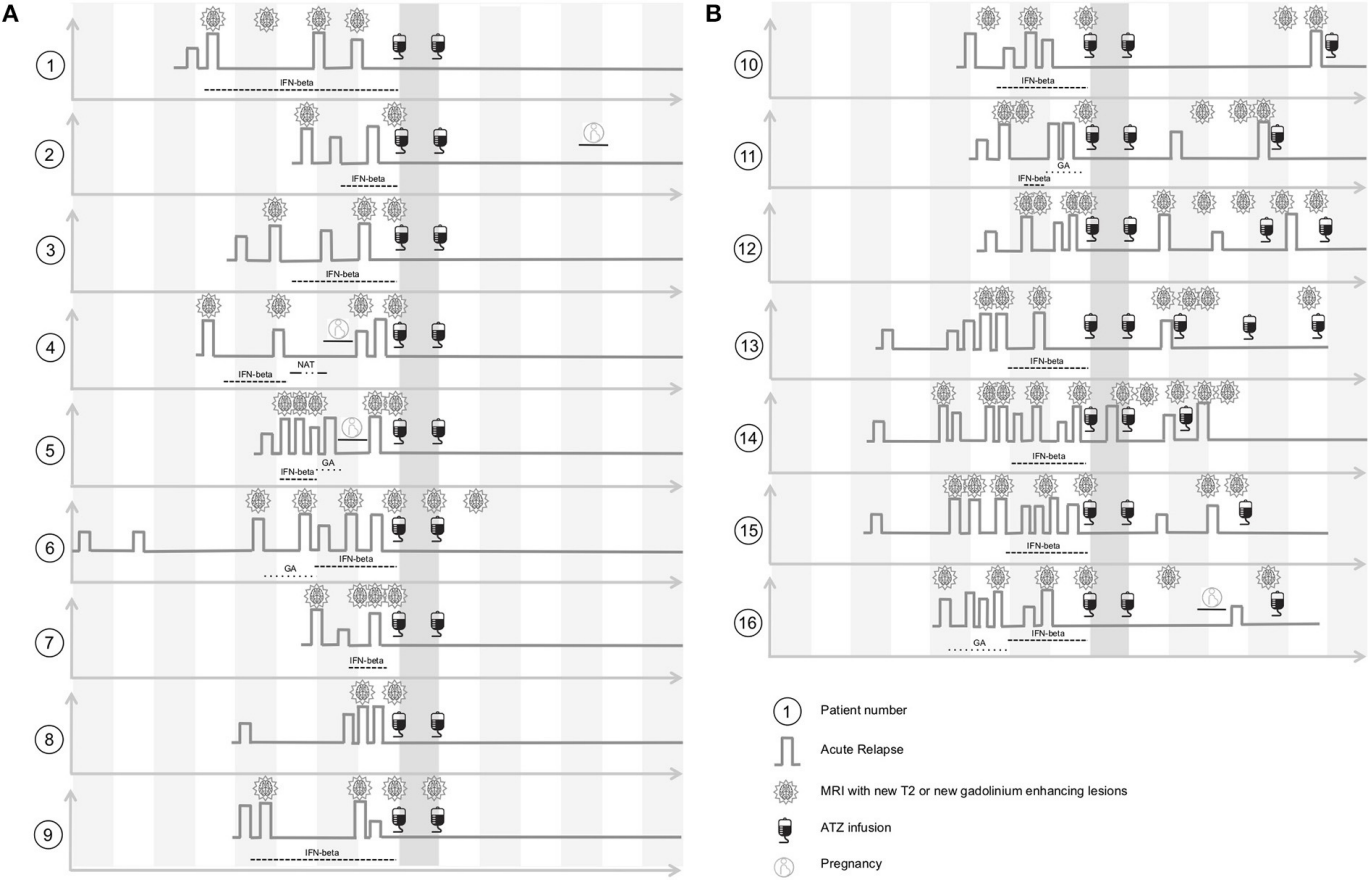
Clinical characteristics of ATZ treated patients before and after ATZ infusion. **(A)** ATZ treated patients without ATZ retreatment are depicted (complete-responder, patient 1–9). Clinical disease course including pre-treatment, relapse activity and MRI progress within yearly MRI scans before and after ATZ infusion are shown threw yearly time intervals. After ATZ infusion, follow up evaluation up to 7 years are presented. **(B)** ATZ treated patients that were retreated with ATZ additionally to two standard ATZ treatments are depicted (partial-responder, patient 10–16). Clinical disease course including pre-treatment, relapse activity and MRI progress within yearly MRI scans before and after ATZ infusion and intervals of ATZ retreatment are shown threw yearly time intervals. After ATZ infusion, follow up evaluation up to 7 years are presented. GA, glatiramer acetate; IFN-beta, interferon-beta; NAT, natalizumab.

We conducted a detailed follow-up of 16 patients with active RRMS (Figure 1). Patients commenced ATZ therapy during the CARE-MS1/2 trial between 2008 and 2011 and were evaluated for up to 7 years until 2017. The immunological sub-analysis was approved by the institutional review board of the University Hospital of Dresden. Patients gave written informed consent that was obtained according to the Declaration of Helsinki.

### Infusion Protocol and Blood Sampling

All patients were treated in accordance with the standard, predefined treatment scheme utilized in the CARE-MS 1 and 2 clinical trials. During the first course, ATZ 12 mg was infused on five consecutive days. Twelve months later a second course was applied on three consecutive days. Blood samples were drawn before the first course (Baseline, month 0) and every 3 months thereafter for up to 7 years. After collection of heparinised blood samples, peripheral blood mononuclear cells (PBMC) were prepared by Ficoll-Hypaque (Biochrom, Berlin, Germany) density centrifugation following the manufacturer's instructions. Cells were frozen in fetal calf serum (FCS, Gibco) with 10% DMSO (Sigma-Aldrich, St. Louis, US-MO) using controlled-rate freezing containers (Nalgene Nunc Int., Rochester, US-NY). Cells were stored in liquid nitrogen prior to analysis.

In parallel, clinical and MRI data including EDSS and relapse activity were collected prospectively. Clinical parameters were evaluated by a Neurostatus-certified neurologist. MRI was conducted every 12 months. Clinically confirmed relapses were treated with i.v. corticosteroids. In case of clinical disease activity or progression on MRI (new gadolinium enhancing lesions and/or two T2 lesions), ATZ re-treatment with an additional course of ATZ 12 mg given on three consecutive days was considered (Figure 1).

### Immune Cell Phenotyping by Fluorescence-Activated Cell Sorting (FACS)

Frozen PBMC were defrosted and washed with culture medium before further preparation. Subpopulations of T cells, B cells, natural killer (NK) cells and antigen-presenting cells (APC) were characterized by surface staining with fluorescence labeled anti-CD3, anti-CD4, anti-CD5, anti-CD8, anti-CD14, anti-CD16, anti-CD19, anti-CD27, anti-CD38, anti-CD39, anti-CD45RA, anti-CD45RO, anti-CD56, anti-CD127, anti-CD138, anti-HLADR, anti-BDCA1, anti-BDCA2, anti-slan (Miltenyi Biotec, Bergisch-Gladbach, Germany) according to the manufacturer's instructions. Negative controls included directly labeled or unlabeled isotype-matched irrelevant antibodies (BD Biosciences). For additional characterization of intracellular markers PBMCs were suspended in culture medium consisting of RPMI 1640 (Biochrom), 5% human AB serum (CC pro, Neustadt, Germany), 2 mM L-glutamine, 100 U/ml penicillin and 100 μg/ml streptomycin (Biochrom). To evaluate cytokine release and T cell polarization, PBMC were stimulated with 10 ng/ml phorbol myristate acetate (PMA, Sigma-Aldrich) and 1 μg/ml ionomycin (Sigma-Aldrich) in the presence of 0.2 μM Monensin (Biomol, Hamburg, Germany) for 6 h prior to analysis. Before characterization of intracellular markers, cells were fixed with fresh prepared fixation concentrate, and permeabilized with wash-permeabilisation concentrate (Fixation/Permeabilisation Buffer Set, eBioscience). Subsequently, cells were stained using fluorescence labeled anti-interferon-gamma, anti-IL-17A (BioLegend, London, UK), or anti-FoxP3 antibody (Miltenyi Biotec) or isotype-matched irrelevant antibody (BD Biosciences). After the staining procedure, cells were evaluated by flow cytometry. Cells were measured on a LSR-Fortessa (BD Biosciences) and evaluated by FACS-Diva Software (BD Bioscience).

### Statistical Analysis

Data were analyzed using a Generalized Linear Mixed Models with Gamma distribution and log link function due to the right-skewed distribution pattern of the data. For the analyses of pre-relapse phases, doses of ATZ were included as random factor. Bonferroni correction for pairwise tests was used. Values of *p* < 0.05 were considered significant. Kaplan-Meier estimates were provided for relapse-free survival (RFS). The length of the comparable time segment (CTS) for comparisons between subjects with and without relapses will be the maximum number of months between the second ATZ course and the first relapse that occurred in the study population. The start of the CTS will be the respective number of months prior to the first relapse of a subject or, for relapse-free subjects, under stable conditions after the second ATZ course (defined as 12 months after the second ATZ course or 24 months after the initial treatment). Receiver Operating Characteristic (ROC) curves and respective Areas under the Curve (AuC) were calculated comparing the ability of potential predictors to classify between stable event-free subjects and subjects with an upcoming relapse (estimated by the differences of the parameters between the start and the end of the CTS period). All statistical analyses were performed using the IBM SPSS Software for Windows (Version 25.0; IBM Corporation, Armonk, NY, USA).

## Results

### Clinical Characteristics of the Long-Term ATZ Cohort

Sixteen patients (11 female, 5 male; average age 30.1 +/− 7.5 years) were included in our observational sub-study and evaluated for up to 7 years' follow up (Figure 1). Prior to ATZ treatment, 13 patients were treated with injectables, one patient received natalizumab, and two patients were treatment naive (Figure 1). All patients suffered from an active disease course at the time of ATZ initiation, defined by relapse and MRI activity 12 months prior (Figure 1). Mean EDSS at ATZ start was 2.5 (+/− 1.3). After the first ATZ infusion course, EDSS score improved on average about 0.5 points and remained stable during long-term follow up. Nine out of 16 patients presented with stable disease without re-appearance of clinical or MRI disease activity, even at 7 years follow up [defined as “complete-responder” (CR), Figure 1, patients 1–9]. Due to recurrence of clinical and MRI disease activity, 7 patients received additional ATZ courses (“partial-responders” (PR), Figure 1, patients 10–16). Disease activity was defined by clinical relapses and/or subclinical MRI progression (new gadolinium enhancing lesions or appearance of two or more new T2 lesions in yearly MRI scans). One of the CR and one of the PR patients became pregnant after the second course of ATZ (Figure 1).

### Depletion and Repopulation Pattern of T Lymphocyte Subsets in ATZ Complete-Responder Patients

**Figure 2:**
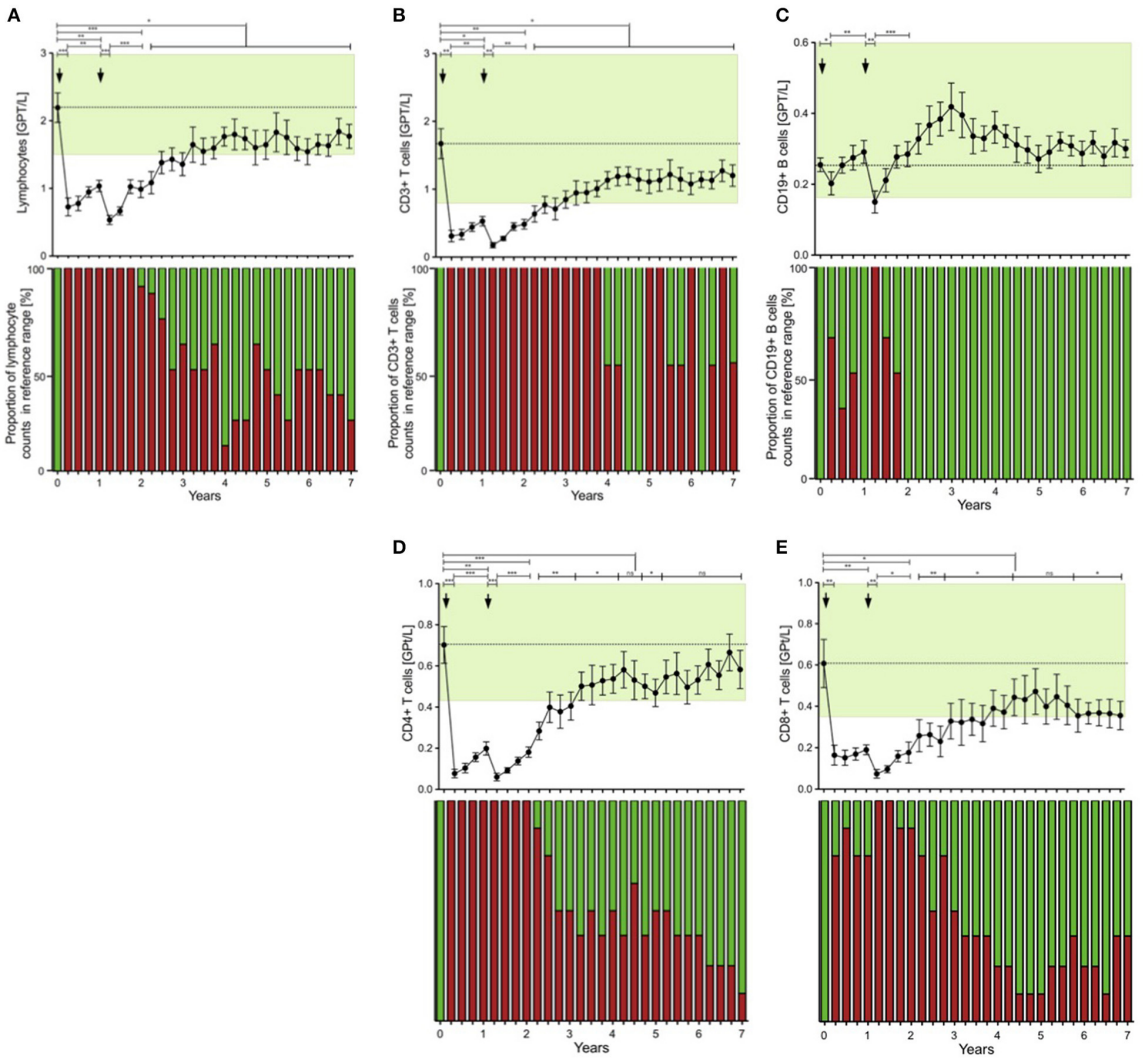
Distribution of peripheral lymphocyte subsets of patients with stable disease course after 1st and 2nd ATZ (complete-responder). Distribution of absolute count of lymphocytes **(A)**, CD3+ T cells **(B)**, CD19+ B cells **(C)**, CD4+ T cells **(D)** and CD8+ T cells **(E)** before (0 years) and up to 7 years follow up are depicted. Mean values +/− SD of lymphocytes and its subsets at each of the 3 monthly evaluations are shown. Reference ranges are marked by pastel green color and baseline values are highlighted by the dotted line. Additionally, proportion of patients that reached values in reference range (green) vs. not (red) are presented for each individual time point and immune cell subtype. Asterisks indicate a statistically significant difference (*p < 0.05, **p < 0.01, ***p < 0.001).

Before commencement of ATZ, all of the CR patients had white blood cell counts with lymphocyte subsets in their normal physiological range (Figure 2). Lymphocyte counts dropped after the first and second ATZ courses, followed by repopulation. However, none of the patients reached their reference range before month 21, and baselines level were not reach until at least month 27 (Figure 2A). At year 3, half of the treated patients had lymphocyte counts back in the physiological reference range (Figure 2A). There were no patients with lymphocyte counts lower than 1.0 GPT/L (Figure 2A). Most of the patients demonstrated lower lymphocyte counts than baseline even after 7 years follow up (Figure 2A).

**Figure 3:**
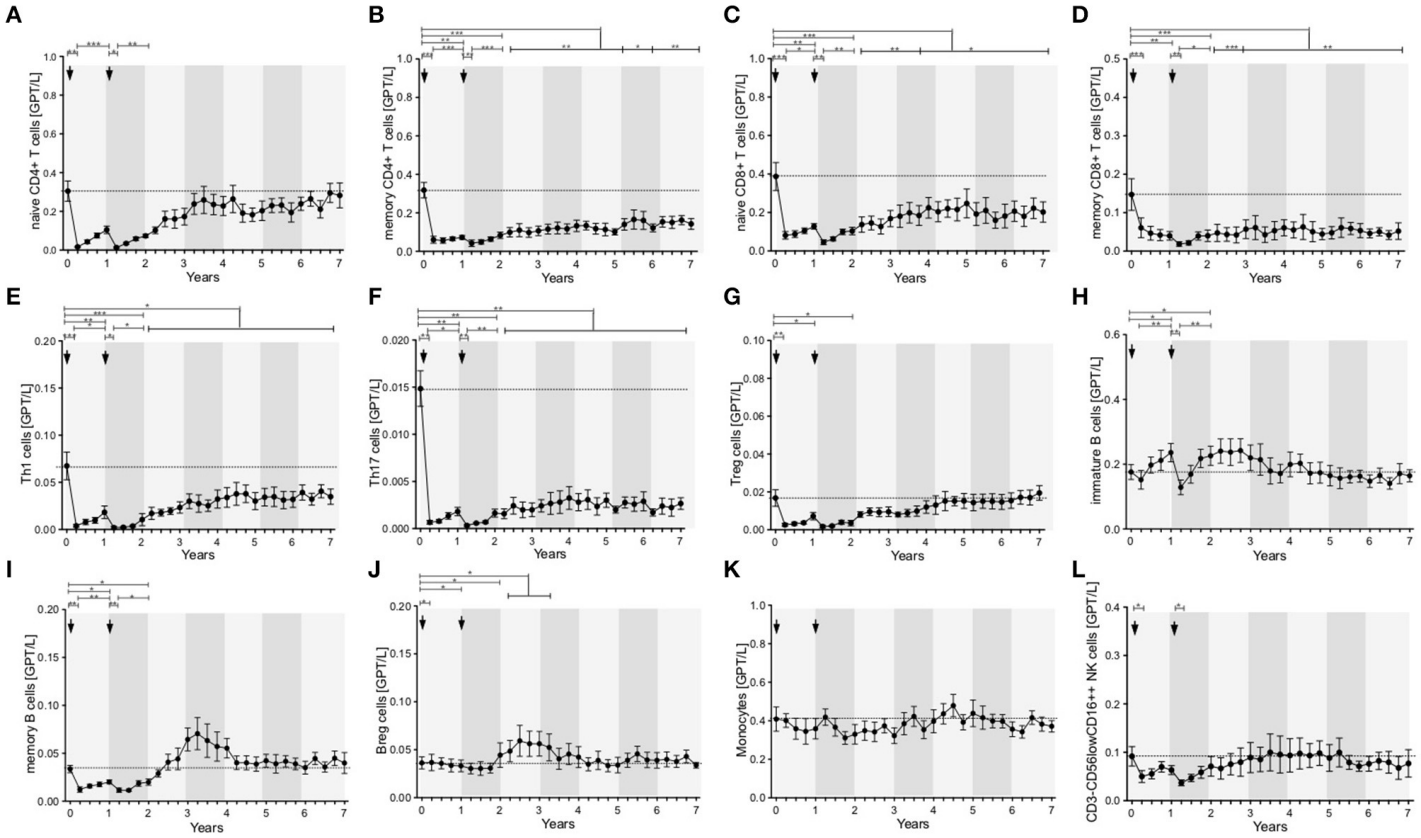
Distribution of peripheral immune cell subsets of patients with stable disease course after 1st and 2nd ATZ (complete-responder). Distribution of absolute count of peripheral immune cell subsets before (0 years) and up to 7 years follow up are depicted. Mean values +/− SD of absolute count of T cell **(A–G)**, B cell **(H–J)**, and innate immune cell **(K,L)** subsets are shown. Asterisks indicate a statistically significant difference (*p < 0.05, **p < 0.01, ***p < 0.001).

**Figure 4:**
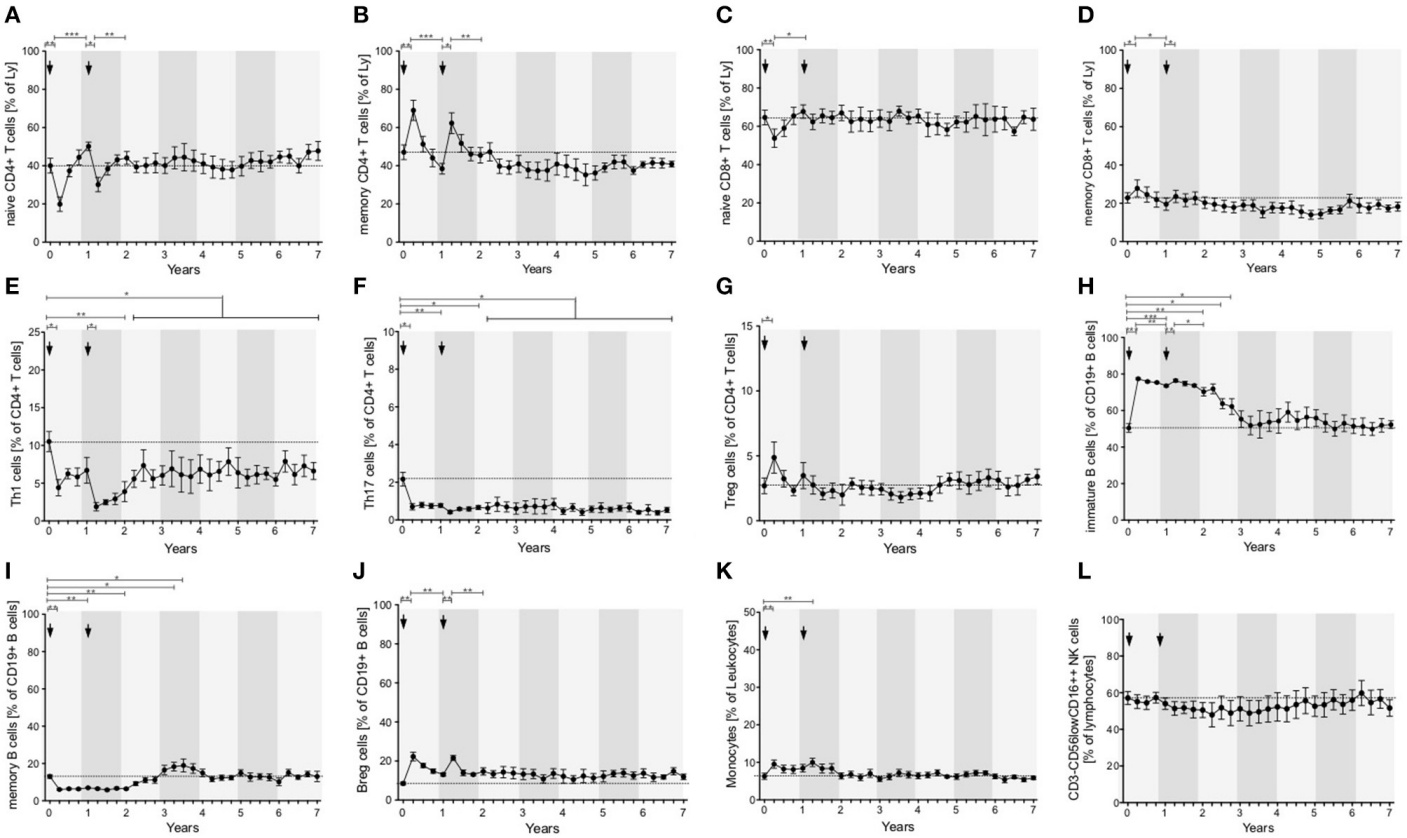
Distribution of peripheral immune cell subsets of patients with stable disease course after 1st and 2nd ATZ (complete-responder). Distribution of relative count of peripheral immune cell subsets before (0 years) and up to 7 years follow up are depicted. Mean values +/− SD of relative count of T cell **(A–G)**, B cell **(H–J)**, and innate immune cell **(K,L)** subsets are presented. Arrows indicate ATZ application. Asterisks indicate a statistically significant difference (*p < 0.05, **p < 0.01, ***p < 0.001).

Lymphocytes (especially CD3^+^) and CD4^+^ and CD8^+^ T cells were markedly depleted after both ATZ courses (Figures 2B,D,E). Although repopulation was seen for all these subpopulations, all T cell counts were significantly lower than baseline over 7 years of observation (Figures 2B,D,E). Among CD4^+^ and CD8^+^ T cell subtypes, naïve and memory CD4^+^ and CD8^+^ T cell counts decreased in absolute terms (Figures 3A–D). Examining relative cell counts after both ATZ courses, the proportion of naïve T cell subtypes was reduced, in contrast to an increase in the proportion of memory T cells (Figures 4A–D).

In addition, T cell subsets have were analyzed by functional characterization using intracellular FACS staining: IFN-gamma producing Th1 resp. IL-17 releasing Th17 CD4^+^ T cells were analyzed. Both subsets were rapidly depleted after ATZ (Figures 3E,F, 4E,F). Repopulation and increase of Th1 cells occurred slowly and did not reach baseline level within the 7-year evaluation (Figure 3E). After ATZ courses, pro-inflammatory Th17 cell counts remained decreased both in absolute and relative terms, and remained lower than baseline levels during the entire observation period in all patients with stable disease course (Figures 3F, 4F). Evaluation of Treg cells marked a relative increase 3 and 6 months after both ATZ courses (Figure 4G), although absolute Treg count decreased (Figure 3G). In contrast to pro-inflammatory T cell subsets, Treg cells constantly repopulated, expended and reach absolute baseline values at year 3 (Figures 3G, 4G). There were no specific patterns related to the baseline levels, degree of depletion or repopulation for either complete lymphocyte count or lymphocyte subsets.

### Depletion and Repopulation Pattern of B Lymphocyte Subsets in ATZ Complete-Responder Patients

In parallel to the changes described above, absolute cell number of CD19^+^ lymphocytes underwent a rapid decrease after ATZ (Figure 2C). In contrast to T cells, CD19^+^ lymphocyte count reached reference range in most of the treated patients in just 3 months post-ATZ (Figure 2C). Absolute CD19^+^ B cell counts overshot baseline levels by up to 3-fold between years 2 and 4 (Figure 2C). This was particularly evident in immature B cells that underwent rapid hyper-repopulation following initial depletion (Figure 3H). Relative counts of immature B cells significantly increased in all treated patients and declined to baseline levels after year 3 (Figure 4H). Absolute and relative counts of memory B cells showed a marked decrease after first ATZ course and remained depressed until the second course (Figure 4I). An overshot in absolute memory B cell count was seen between years 3 and 4 (Figure 4I). In common with observations for Treg cells, Breg cells were also relatively increased in number 3–6 months after ATZ infusions, and remained stable around baseline values for up to 7 years (Figure 3J). Periods of B cell hyper-repopulation were not associated with reappearance of MS disease activity in the patients. Six patients developed autoimmune hyper- or hypo-thyroidism years after ATZ infusions, which was not correlated with specific B cell profiles.

### Depletion and Repopulation Pattern of Innate Immune Cells in ATZ Complete-Responder Patients

After both ATZ courses, absolute count of monocytes and corresponding subsets (classical monocytes, intermediate and non-classical monocytes) were not significantly affected (Figures 3K, 4K). Due to relevant decrease in lymphocyte number, relative count for monocytes increased after each ATZ infusion but declined and remained stable around baseline levels during follow up (Figure 4K). There was a transient increase in relative but not absolute number of DC populations including slanDC, BDCA1^+^ DC and BDCA2^+^ DC. In contrast, absolute NK cell count was decreased up to 12 months after each ATZ infusion course (Figure 3L). Within NK cell subsets, CD3-CD56lowCD16^++^ NK cells were primarily affected by ATZ and decreased in number (Figures 3L, 4L), whereas numbers of CD3-CD56^++^CD16- NK cells and CD3-CD56^+^CD16^−^ NK cells were not significantly changed. Twelve months after the second ATZ course, NK cells reached baseline levels and remained stable over the 7 year follow up (Figures 3L, 4L).

### Depletion and Repopulation Pattern of Peripheral Immune Cell Subsets in ATZ Partial-Responder Patients

Seven patients presented with reappearance of clinical and MRI disease activity (ATZ partial-responders, PR) in the follow up period (Figure 1, patients 10–16). Based on the ATZ retreatment protocol, ATZ re-dosing was conducted in cases of emergent disease activity defined by acute relapses or MRI activity−10 ATZ retreatments have been undertaken (Figure 1).

**Figure 5:**
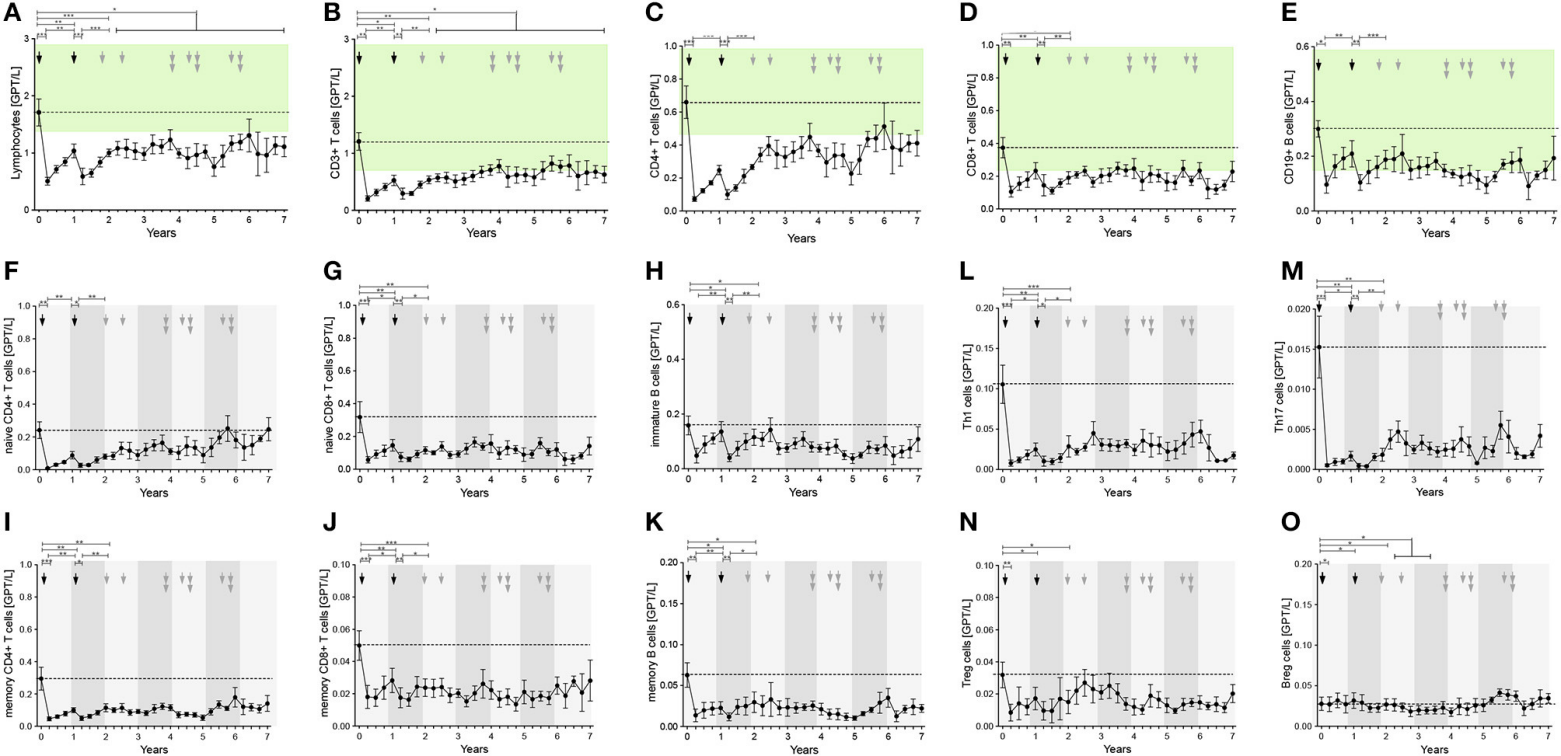
Distribution of peripheral immune cell subsets in patients with reappearance of disease activity after 1st and 2nd ATZ (partial-responder). Distribution of absolute count of peripheral immune cell subsets before (0 years) and up to 7 years follow up are depicted. Mean values +/− SD of absolute count of lymphocytes **(A)**, T cell **(B–D, F,G, L–J, N)** and, B cell **(E,H,K,O)**, subsets are shown. Reference ranges are marked by pastel green color and baseline values are highlighted by the dotted line. Arrows in black indicate 1st and 2nd ATZ infusions, arrows in gray indicate time point of ATZ retreatment in individual patients. Asterisks demonstrate statistical significance (*p < 0.05, **p < 0.01, ***p < 0.001).

The first approach to evaluate depletion and repopulation of peripheral immune cell subsets in the PR group was analyzed on group level using mean values (Figure 5). Baseline evaluation of peripheral immune cell subsets presented levels in a similar range to the CR cohort (Figures 5A–E). In all patients, normal depletion of T and B cell subsets after ATZ courses took place (Figures 5A–E). In the first months after ATZ courses, repopulation of peripheral immune cell subsets did not differ between responder groups (Figure 5). Within the PR group, ATZ retreatment was done at the different time points marked by gray arrows in Figure 5. Compared to the CR group, depletion and repopulation in the PR group featured higher variability especially in CD4^+^ T cells, Th1 cells, Th17 cells, and B cell subsets (Figure 5). In line with the data for the CARE MS 1 and 2 study cohort using evaluations on group level (17), CR and PR patients receiving ATZ could not be differentiated by immunoprofiling.

### Innovative Approach With Event-Driven Analysis of Immunoprofiling in ATZ Partial-Responder Patients

**Figure 6:**
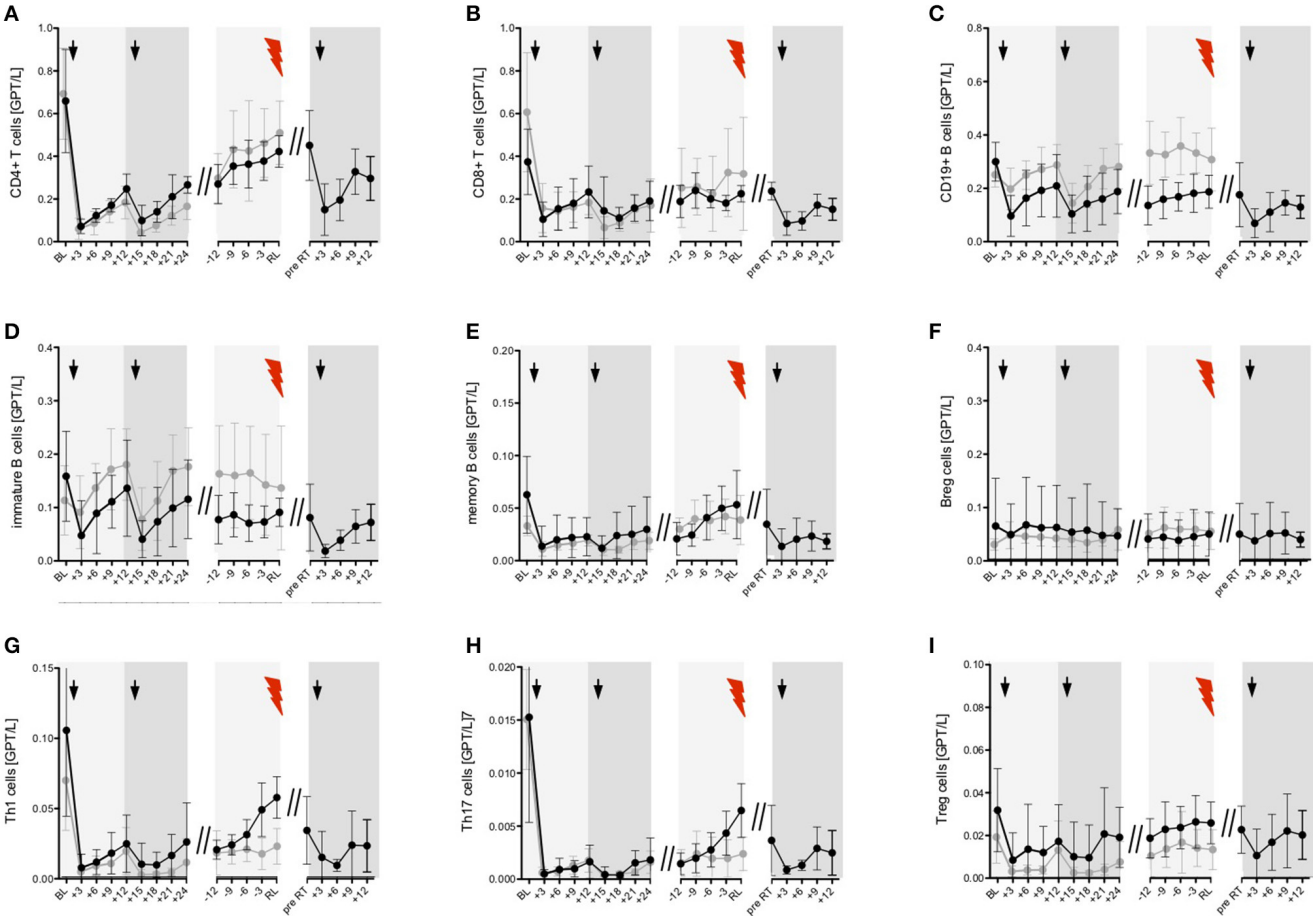
Event-driven immune cell phenotyping before relapse activity and after ATZ retreatment. Distribution of absolute immune cell count of selected T **(A,B, G–I)** and B cell **(C–F)** subsets are presented. Black line—partial-responder patients: Immune cell subsets before (baseline, BL) and after 1st and 2nd ATZ infusion 3 monthly up to 24 months follow up (+3, +6, +9, +12, +15, +18, +21, +24) are depicted. Mean values +/− 95%CI of all partial-responder patients are shown. Additionally, distribution of absolute immune cell count before clinical appearance of an acute relapse (RL, red flash) was evaluated in 3 monthly intervals (−12, −9, −6, −3). Mean values +/− 95%CI of 13 relapses are presented. Furthermore, absolute immune cell count before ATZ retreatment (pre RT) and during 3 monthly follow up (+3, +6, +9, +12) are demonstrated. Mean values +/− 95%CI of 10 ATZ retreatments are shown. Gray line—complete-responder patients: Immune cell subsets before (baseline, BL) and after 1st and 2nd ATZ infusion are depicted. In addition, period of stable disease course 24 months after 2nd ATZ infusion are presented. Mean values +/− 95%CI of all complete-responder patients are shown. Arrows indicate ATZ infusion.

In 7 of 16 ATZ treated patients, 13 relapses have been confirmed and treated with i.v. corticosteroids (Figure 1B). On average, the first relapses in the PR group appeared 39 +/− 6.6 months (mean +/− SD) after first ATZ course. PR patients received up to three ATZ retreatments—in total, 10 additional ATZ courses have been applied (Figure 1B). To analyze the individual patterns in data from PR patients demonstrating disease activity, our immunological analysis was conducted using an event-driven approach related to the onset of relapse activity and the time-point of retreatment (Figure 6). Depletion and repopulation patterns related to the two ATZ courses were not different between the PR and CR groups (Figure 6). Analyzing the time period 12 months before the MS relapse in an event-driven manner, CD4^+^ T cells, CD8^+^ T cells, CD19^+^ B cells, as well as naïve and memory T cell subsets and Treg cells, immature B cells and B reg cells, were stable and did not significantly differentiate CR patients from PR patients (Figures 6A–D,F,I). Analyzing pro-inflammatory T cells via event-driven analysis, we could demonstrate an absolute increase of Th1 and Th17 cells starting 9 months before relapse and peaking at the relapse itself (Th1 cells 0.069 (0.038–0.124), Th17 cells 0.004 (0.002–0.007), [mean in GPT/l (95% CI)]; (Figures 6G,H)]. Compared to the prior steady state period (12–18 months before relapse), this relapse-associated absolute increase was ~0.041 +/− 0.022 GPT/L (mean +/− SD) for Th1 cells and ~0.0055 +/− 0.0029 GPT/L for Th17 cells. In contrast, CR patients persistently showed low absolute levels of Th1 and Th17 cells during the whole period of clinical stable disease (Th1 cells 0.019 (0.011–0.035), Th17 cells 0.002 (0.001–0.003), [mean in GPT/l (95% CI); (Figures 6G,H). In CR patients, intra-individual variability of Th1, respectively, Th17 cells during clinical stable disease after months 24 (calculated as the mean standard deviation of absolute cell counts assessed every 3 months in each patient) presented at 0.017 (+/− 0.007) GPT/L for Th1 cells resp. 0.0018 (+/− 0.0009) GPT/L for Th17 cells. This variability was significantly lower than the described increase prior to relapse in the PR group.

A further analysis using ROC curves and corresponding AUC demonstrated that evaluation of Th1 cell increase (AUC = 0.833; *p* < 0.05) resp. the combined evaluation of Th1 plus Th17 cell increase (AUC = 0.854; *p* < 0.05) were the best predictive cell markers to classify between clinically stable subjects and upcoming relapse activity.

In addition to the event-driven Th1 and Th17 changes, there was a small albeit insignificant increase observed in the memory B cell counts starting 6 months before relapse, with highest values at clinical onset of the relapse itself (Figure 6E). After ATZ retreatment of PR patients, counts for all evaluated T and B cell subsets again rapidly decreased (Figure 6).

## Discussion

The monoclonal anti-CD52 antibody ATZ led to rapid and dramatic depletion of various lymphocyte subsets (7, 8). Although hypothesized previously, we confirmed that the depletion and repopulation profile is not only defined by cellular CD52 expression, but also by other factors such as susceptibility to ATZ-mediated antibody and complement-dependent cytotoxicity (18, 19). After the initial cellular depletion, immunological reprogramming including recovery and reorganization of immune cell subsets are assumed to be essential for permanent remission in MS—up to now, this has been demonstrated in the majority of CARE MS 1 and 2 patients. Despite these clear cellular changes, there currently no biomarkers which can predict duration of response to ATZ treatment and determine necessity for ATZ retreatment (20).

In our cohort, all patients responded to a standard two-course ATZ infusion procedure consisting with decreases in clinical and MRI disease activity. While some patients demonstrated “complete-response” with NEDA-3 status and no need for additional ATZ, others were characterized by “partial responses” that featured reappearance of disease activity some years after initial ATZ infusions.

In all patients, ATZ lead to marked depletion followed by repopulation of CD4^+^ and CD8^+^ T cell subsets over the 7 years of the study, confirming previous evidence of short T cell repopulation intervals (9, 10). Patients did not reach baseline levels for CD3^+^, CD4^+^, and CD8^+^ lymphocyte subsets in the study period. The pattern of baseline level, degree of depletion and repopulation of CD4^+^ and CD8^+^ T cells could not predict ATZ efficacy or reappearance of disease activity in ATZ treated MS patients. This contrasts with animal data where an association has been postulated (13, 21).

Pivotal clinical trials of ATZ have demonstrated that retreatment is needed in <50% of ATZ-treated patients (6). Individual MS disease activity, differences in pre-treatment and differences in individual immunological profiles have been associated with complete or only partial response in ATZ treated patients (20). Evaluating mean numbers of lymphocytes subsets over time, we could not find a systematic difference in the immune-profiling between ATZ CR vs. PR patients. Our results are in line with other reports focusing on pooled analyses of immunological data of patients with or without disease activity (13, 15). Due to the fact that the timing of reappearance of disease activity was individually different from patient to patient, we implemented an event-driven approach to analysis in our cohort. We present an individual, relapse-focused analysis of T and B cell subsets in ATZ patients. Th1 and Th17 cells are known to be important pro-inflammatory players in MS pathology that perpetuate the inflammatory processes, particularly the in CNS (22, 23). Additional data indicate that functional impairment of Treg cells in MS enables activation of pro-inflammatory Th1/Th17 cell responses in MS (24, 25). These cells can normally inhibit auto-reactive Th1 and Th17 cells, and thereby limit inflammatory immune responses (26, 27). Although Treg cells are generally decreased in numbers after a depletion course, ATZ treatment induces a Treg cell phenotype within CD4^+^ T cells, that was also observed in our cohort (9, 28). After the second ATZ course, Treg cell counts recovered while pro-inflammatory Th1 and Th17 cells remained repressed after depletion, especially in the CR group. The restored long-term, immunological balance between Th1/Th17 and Treg cells may explain the long-term efficacy of ATZ in patients defined as CR. In the PR group, reappearance of clinical activity was associated with an increase of absolute Th1 and Th17 cell count. Although initially depleted comparable to CR patients, Th1 and Th17 cells steadily increased 9–6 months before onset of relapse and peaked at the clinical appearance of the relapse itself. In line with our new cellular functional data, Mercanti et al. previously reported that IL17 mRNA was significantly elevated in ATZ patients with relapses compared to patients without clinical disease activity (29). Nevertheless, these data have not been linked to timing of the clinical event. Therefore, since the present study on cellular markers includes information on timing, the increase of absolute Th1 and Th17 numbers seems may be the most promising predictor for relapsing disease after ATZ treatment.

Comparable with data from other studies, CD19+ B lymphocytes in the present study recovered quickly in just 3 months after first ATZ course –T cells remained reduced for longer (10, 11, 14). After the second ATZ course, recovery in terms of delayed hyper-repopulation was found in all investigated B cell subtypes, although baseline values were eventually reached. Previous reports introduced the hypothesis of a B cell overshot associated exacerbation of CNS inflammation after ATZ treatment whereas others reported that ATZ is highly effective in B cell mediated MS (30, 31). However, in our cohort, although the overshot was also observed, the B cell repopulation was not associated with disease exacerbation. Among B cell subsets, immature B cells showed the highest fluctuations with depletion and hyper-repopulation after first and second ATZ course (14). Decrease of B cell numbers during ATZ depletion in peripheral blood is more intense compared to lymphoid organs and bone marrow (32, 33). Repopulation of immature and naïve B cells from precursor cells of bone marrow is thought to be responsible for the delayed overshot of B cell recovery after ATZ (2, 14, 34). Previous reports discussed prolonged altered phenotype and long-term shift to immature B cell subsets after ATZ that may contribute to efficacy in MS (2, 14). Further studies point to the marked and long-lasting depletion of memory B cells after ATZ (10, 14). This is in line with our data whereby we demonstrate that memory B cell counts are decreased for at least 2 years after first ATZ course. Memory B cells are proposed regulators of autoimmunity, and are associated with autoimmune disease activity (34–37). Several disease modifying MS treatment regimes are notable for their impact on memory B cells (37–40). Decrease in memory B cell counts has been linked to a stable disease course after ATZ treatment in MS (10, 14). We could demonstrate that reappearance of clinical MS activity was accompanied by an increase in memory B cell counts, whereas immature B cell subsets did not show significant changes. Similar to the changes of Th1 and Th17 cell counts related to clinical activity, increase of absolute memory B cell numbers started 9–6 months before onset of relapse. As there was also a constant increase and hyper-repopulation of memory B cells after ATZ course in the CR group, our data could not statistically differentiate the kinetics of memory B cells between PR and CR in our event-driven analysis with this small samples size. Therefore, in contrast to the pro-inflammatory T cell subsets, memory B cells cannot serve as a clear predictor for up-coming disease activity in our small pilot study.

Recent reports demonstrated, that cells of the innate immune system were transiently impaired but rapidly rebalanced and fully restored in function after initial ATZ course (8, 32). This has been further evidenced in our long-term follow up over 7 years whereby absolute counts of different DCs, monocytes and NK cell subsets in the peripheral blood remained stable during the entire evaluation period. These data may explain the low incidence of serious infections and the recovered level of immunocompetence that are evident years after ATZ treatment (6).

With only 16 patients evaluated in our cohort, our study lacks the power to generate specific cut-offs for respective markers. A further limitation of our study is difficulty in defining the lengths of comparable time segments between both groups which was necessary for the statistical analysis between the two groups. Thus, in our study we are only able to define a hypothesis regarding event-associated cellular markers that may predict upcoming disease activity during ATZ treatment. Although the patient group was small, we could demonstrate clear and significant findings on Th1 and Th17 cells as markers for upcoming disease activity. Studies with higher samples size would enable sophisticated statistical analysis and are now essential to clarify the relevance of additional peripheral immune cell subsets (e.g., memory B cells) and their potential to predict disease activity during ATZ treatment.

In summary, our data confirm for the first time the long-term immunological response in ATZ treated patients with a durable decreases in Th1/Th17 CD4^+^ cell counts after standard courses of ATZ. Our results indicate that reappearance of clinical disease activity is associated with an increase in Th1 and Th17 cell populations. Event-driven approaches to immunoprofiling analysis should be applied in larger datasets of clinical studies to confirm our findings. Frequent monitoring of lymphocyte subsets may assist in decision-making of ATZ re-dosing in clinical practice.

## Data Availability Statement

The datasets generated for this study are available on request to the corresponding author.

## Ethics Statement

The studies involving human participants were reviewed and approved by the institutional review board of the University Hospital of Dresden (Ethikkommission an der Technischen Universität Dresden). The patients/participants provided their written informed consent to participate in this study.

## Author Contributions

KA and TZ: study concept and design. KA, JB, and MM: acquisition of data. KA, JB, and RH: analysis and interpretation of data. KA and TZ: drafting of the manuscript. JB and TZ: critical revision of the manuscript for important intellectual content. RH and KA: statistical analysis. TZ: study supervision.

### Conflict of Interest

KA received personal compensation for oral presentations and consulting services from Biogen Idec, Merck, Sanofi, and Roche. TZ received personal compensation from Almirall Biogen, Bayer, Celgene, Novartis, Roche, Sanofi, and Teva for consulting services. TZ received additional financial support for research activities from BAT, Biogen, Novartis, Roche, Teva, and Sanofi Aventis. RH received speaker fees from Sanofi. The remaining authors declare that the research was conducted in the absence of any commercial or financial relationships that could be construed as a potential conflict of interest.

## Abbreviations

APC: antigen-presenting cells
ATZ: alemtuzumab
AUC: Areas under the Curve
CR: complete-responder
CTS: comparable time segment
DC: dendritic cells
EDSS: expanded disability status scale
FACS: fluorescence-activated cell sorting
FCS: fetal calf serum
IFN-gamma: interferon-gamma
IL: interleukin
MRI: magnet-resonance imaging
MS: multiple sclerosis
NEDA: no evidence of disease activity
NK cell: natural killer cell
PBMC: peripheral blood mononuclear cells
PR: partial responder
RFS: relapse-free survival
ROC: Receiver Operating Characteristic
RRMS: relapsing-remitting multiple sclerosis.
